# Expediting telehealth use in clinical research studies: recommendations for overcoming barriers in North America

**DOI:** 10.1038/s41531-021-00177-8

**Published:** 2021-04-12

**Authors:** Anna Naito, Anne-Marie Wills, Thomas F. Tropea, Adolfo Ramirez-Zamora, Robert A. Hauser, Davide Martino, Travis H. Turner, Miriam R. Rafferty, Mitra Afshari, Karen L. Williams, Okeanis Vaou, Martin J. McKeown, Letty Ginsburg, Adi Ezra, Robert Iansek, Kristin Wallock, Christiana Evers, Karlin Schroeder, Rebeca DeLeon, Nicole Yarab, Roy N. Alcalay, James C. Beck

**Affiliations:** 1 Parkinson’s Foundation, Miami, FL USA; 2 Department of Neurology, Massachusetts General Hospital, Harvard Medical School, Boston, MA USA; 3 Department of Neurology, Perelman School of Medicine, University of Pennsylvania, Philadelphia, PA USA; 4 Department of Neurology, Norman Fixel Institute for Neurological Diseases, University of Florida, Gainesville, FL USA; 5 Department of Neurology, University of South Florida, Tampa, FL USA; 6 Department of Clinical Neurosciences, University of Calgary, Calgary, AB Canada; 7 Department of Neurology, Medical University of South Carolina, Charleston, SC USA; 8 Shirley Ryan AbilityLab Chicago, Chicago, IL USA; 9 Department of Physical Medicine and Rehabilitation, Feinberg School of Medicine, Northwestern University, Chicago, IL USA; 10 Department of Neurology, Rush University Parkinson’s Disease and Movement Disorders Program, Chicago, IL USA; 11 Department of Neurology, Feinberg School of Medicine, Northwestern University, Chicago, IL USA; 12 Department of Neurology, Steward Medical Group, Brighton, MA USA; 13 Djavad Mowafaghian Centre for Brain Health, University of British Columbia, Vancouver, BC Canada; 14 Division of Parkinson Disease and Movement Disorders, Leonard M. Miller School of Medicine, University of Miami, Miami, FL USA; 15 Department of Neurology, Movement Disorders Unit, Tel Aviv University, Tel Aviv, Israel; 16 School of Clinical Science at Monash Health, Monash University, Melbourne, VIC Australia; 17 Department of Neurology, Columbia University Irving Medical Center, New York, NY USA; 18 Department of Neuroscience and Physiology, NYU Grossman School of Medicine, New York, NY USA

**Keywords:** Conferences and meetings, Clinical trials

## Abstract

Despite data supporting the rapid adoption of telehealth in the delivery of clinical care in North America, the implementation of telehealth visits in clinical research studies has faced critical barriers. These challenges include: (1) variations in state licensure requirements for telehealth; (2) disparities in access to telehealth among disadvantaged populations; (3) lack of consistency among individual Investigational Review Boards (IRBs). Each barrier prevents the systematic conversion of research protocols to include telehealth visits. The Parkinson’s Foundation and members of the Parkinson Study Group submit this Comment to highlight current challenges to implementing telehealth visits for clinical research studies. Our objective is to provide a consensus statement emphasizing the urgent need for regulators to standardize adoption of telehealth practices and to propose recommendations to reduce the burden for implementation in existing research study protocols.

Clinical research studies in the United States (US) came to an abrupt halt in March 2020, due to the COVID-19 pandemic. This momentous event caused shifts in the delivery of clinical care, resulting in adoption of technology-based solutions including the use of virtual visits via audiovisual tele-conferencing, or telehealth. However, this adoption was not as seamless for clinical research studies that aimed to convert in-person study visits to telehealth. This Comment summarizes a panel discussion on the use of telehealth for clinical research held during the August 2020 joint annual meetings of the Parkinson’s Foundation Center of Excellence Leadership Conference and the Parkinson Study Group Annual Meeting, which convened 396 leading global healthcare professionals in the movement disorders neurology field. The authors of this Comment represent attendees of the joint meeting and a wide range of perspectives from leading Movement Disorder neurologists, healthcare professionals, clinical study coordinators, Parkinson’s Foundation, and patient advocates.

A recent survey administered between May 13 and June 11, 2020 among People with Parkinson’s disease (PwP) through the mailing lists of the Parkinson’s Foundation and Columbia University Parkinson’s Disease Center of Excellence reported that among the 1342 respondents, only 131 PwP (9.7%) had used telehealth services to receive clinical care prior to the COVID-19 pandemic^1^. In contrast, 63.5% of PwP (852/1342) reported having used some form of telehealth service during the COVID-19 pandemic (up to June 11, 2020). Among those who had utilized telehealth services during the pandemic, 46% responded that they would prefer to continue using telehealth to receive care post-pandemic^1^. The rapid adoption of telehealth services to deliver clinical care is supported by both the healthcare practitioners and the Parkinson’s disease (PD) community and demonstrates feasibility to deploy these services effectively.

Similar to clinical care, PwP are interested in remote clinical research when available. For example, Tarolli et al. reported that in a sub-study of the multi-center, phase 3 STEADY PD III trial, 95% of participants completed remote video visits^2^. Furthermore, the majority of participants (over 75%) expressed increased likelihood to participate in future clinical research studies if some visits could be conducted remotely^2^. These results indicate a desire among research participants for telehealth utilization and underscores its value for clinical research studies.

Similarly, patient satisfaction for telehealth utilization in Canada has been high and encouraging. There is evidence suggesting that up to 80% of PD patients in some rural areas of Canada, such as Interior British Columbia, would prefer access to telehealth for follow-up neurologist appointments, mainly due to cost and difficulty of travel^3^. Although similar evidence has not been reported specifically for research visits, it is likely that this preference would be consistent, thus indicating the need for a systematic implementation plan at a national, and ideally, global scale.

Despite data supporting the rapid adoption of telehealth in the delivery of clinical care in the US, the implementation of telehealth visits in clinical research studies has faced critical barriers. These challenges include: (1) variations in state licensure requirements for telehealth; (2) disparities in access to telehealth among disadvantaged populations; (3) lack of consistency among individual Investigational Review Boards (IRBs), preventing systematic conversion of research protocols to include telehealth visits.

As sponsors of a multi-center genetic testing clinical research study, PD GENEration, the Parkinson’s Foundation, and members of the Parkinson Study Group jointly submit this Comment to highlight current challenges to implementing telehealth visits for clinical research studies. The objective of this Comment is to provide a consensus statement emphasizing the urgent need for regulators to standardize adoption of telehealth practices and propose recommendations to reduce the burden for implementation in existing research study protocols.

## Barriers for the use of telehealth in clinical research

### Medical licensure requirements using telehealth vary by state/province and institution

With the rapid adoption of telehealth services being offered for clinical care, clinical research studies immediately sought changes to align with the “new norm” of the use of telehealth. Interestingly, the rate of telehealth adoption to conduct research visits has been significantly slower and has posed challenges that are unique to the regulation oversight of clinical research studies. While the Center for Medicare and Medicaid Services in the US allows clinicians to provide clinical care across state lines, i.e., when patients are located in states in which the providers are not licensed, the guideline for clinical research is less clear. In the US, a major barrier to implementing telehealth research visits is the inconsistent interpretations across site IRBs of the state medical licensure requirements to conduct telehealth research visits for study participants who reside in a state where the provider is not licensed. Differences in medical licensure regulations between states makes this interpretation challenging, and a few states have now ratified laws on interstate medical licensure. In the PD GENEration study where participants residing in 21 42 states have been enrolled across six study sites (CA, IL, MA, MN, NY, PA), adapting study protocol assessments from in-person conduct to telehealth resulted in different interpretations and processes by each site IRB. Despite the current federal waiver for state medical licensures to perform telehealth visits for clinical care, when site IRBs were queried about the use of telehealth to perform research visits for out-of-state participants, over 60% affirmed the necessity of medical licensures in the state in which the participant resides to conduct telehealth research visits. Therefore, while medical licensure requirements are regulated at the state level, they are also independently evaluated at the local IRB level, resulting in discrepant implementation across clinical sites.

Similarly in Canada, licensing requirements vary significantly across provinces and encompass the following: (1) four provinces with no licensure requirements to conduct telehealth visits, (2) four provinces waiving licensure only in the context of emergency healthcare, (3) four provinces requiring licensure, and (4) one province with unspecified licensing requirements. The Federation of Medical Regulatory Authorities of Canada is currently working on the development of interjurisdictional licensure for the practice of telehealth. Therefore, jurisdiction of interprovincial use of telehealth for clinical research visits in Canada is still not defined. In contrast, in countries such as Australia and the UK, where medical licensure is regulated by a national board, these challenges are nonexistent. However, Australian regulations recommend recording patient consent, potential confidentiality breach, and inability to fully examine the patient when utilizing telehealth^4^. Interestingly, in Israel, licenses are regulated nationally, however, further guidelines allow providers with global private professional insurance coverage to administer telehealth visits internationally^4^.

In the US, the disparate interpretations of licensure requirements across states pose additional hurdles for navigating legal risk. Thus, providers involved with research and clinical practice are advised to consult with their malpractice insurance policies regarding potential variances in coverage for visits conducted via telehealth.

### Challenges with reaching disadvantaged populations through telehealth

Despite the benefits of reducing participant travel burden, expanded outreach to areas with limited access to health institutions, maximizing safety, and reducing clinic costs (personal protective equipment, sanitization, clinic space: exam room and waiting room, etc.), telehealth equally poses challenges for reaching less technology proficient aging populations and underrepresented minority populations. A 2016 study by the Federal Communications Commission found 39% of Americans in rural areas lacked access to broadband speeds considered “adequate” for “high-quality voice, data, graphics, and video offerings” which they defined as 25 Mps download and 3 Mps upload speed^3,5^. The recent Parkinson’s Foundation survey indicates these challenges may further be augmented by low socioeconomic or educational status^1^. Previous studies have reported that on average, a telemedicine visit could save a participant as much as 3 h of time and 100 miles of travel per visit^6,7^. In the STEADY PD III trial, remote assessments shortened the visit time by about 2 h compared to in-person visits (54 vs 190 min)^2^. While access to transportation is a major limitation circumvented through telehealth, underserved populations also may lack access to technology (devices and connectivity) and have lower technological literacy. At least 20% of households in the US have been reported to lack broadband access at home or have no access to a smartphone^6^. Despite having access to broadband, the feasibility of a telehealth visit is heavily dependent on the speed and quality of the connection. Furthermore, many of these populations may also be susceptible to evolving concerns regarding confidentiality, privacy, and trust.

While best practice approaches to reach underrepresented communities involve in-person engagement, telehealth visits may still be beneficial to improve engagement among these communities. According to the Pew Research Center, there has been a steady increase in the ownership of smartphones among people aged 55 and over from 25% in 2011 to 68% in 2019, demonstrating increased access to reach this population^8^. A few tangible ways to improve engagement through remote methods include involving younger family members who may have greater technological literacy and developing educational materials in native languages to increase accessibility. Access to transportation has been an important historical barrier for people living in rural areas and certain minority populations to participate in clinical research^6^. Telehealth may also allow for more efficient follow-up for study protocols, enhancing compliance while reducing burden for participants and their caregivers. Importantly, developing local partnerships to work alongside these communities will be critical, including local physician groups, medical associations, patient advocacy organizations, and community leaders. In addition, telehealth uniquely presents the potential opportunity for people to be treated remotely by healthcare providers belonging to their own race/ethnicity, which may further improve engagement in clinical research and care^9,10^. Despite technological barriers, the Parkinson’s Foundation’s virtual community engagement programs have reached an audience of over 200,000 people globally. A recent virtual recruitment program delivered in Spanish to support PD GENEration conducted in partnership with the Barrow Neurological Institute amassed over 963 views of the online program and reached 12 Latin American countries.

Some research studies, including those of investigational drugs, are now being designed at the outset, so that PwP can participate virtually without leaving their homes. The Trial of Parkinson’s and Zoledronic Acid is an example of this where participants can consent and confirm their diagnosis virtually, and the study drug is administered by a nurse who comes to their home. This strategy could be leveraged to provide traditionally underrepresented groups with the opportunity to participate in clinical research studies.

### Lack of consistency among Institutional Review Boards

The rapid adoption of telehealth for research studies was seen primarily among clinical sites that already had existing telehealth services, pre-COVID, for routine clinical care. One important lesson learned through these telehealth proficient sites is the development of an a priori institution-wide standard operating procedure (SOP) providing guidelines for the use of telehealth platforms for both clinical and research visits. In addition, some sites had also developed site-specific criteria for the use of electronic consent platforms and other electronic data collection tools such as electronic patient reported outcomes, which greatly helped expedite adaptation of existing protocols to telehealth practices.

While sites that had already developed telehealth SOPs had a review system in-place to quickly adapt existing protocols from in-person to virtual visits, an added challenge for study sponsors was the burden of navigating site-specific requirements that involved bespoke documentation and responses to respective ethics boards. Thus, there is a critical need to develop field-wide criteria that establish minimum thresholds for safety and data/privacy security standards for the use of telehealth platforms in clinical research studies. More importantly, there is a pressing need for systematic implementation of these standards in the field going forward. Despite the issuance of the FDA’s 2016 guidance document on the use of electronic informed consent, sites did not uniformly have processes in place to review and approve the use of these platforms, thus resulting in significant delays for telehealth integration. As of September 2020, the FDA published a Guidance on the Conduct of Clinical Trials of Medical Products During COVID-19 Public Health Emergency. However, it is important to note that IRBs did not uniformly adopt these recommendations in the US. In Canada, the climate of relative uncertainty regarding jurisdiction and medical licensure requirements implicate diversity of interpretation across ethics committees of different sites, creating a situation similar to the one experienced in the US^11^.

Another determination that may help expedite the conversion of study protocols to adapt telehealth use is for ethics boards to consider distinguishing review pathways for interventional studies vs non/minimal-risk observational studies. This approach would allow efficient triaging of study protocol reviews and lower the burden for the need for protocol amendments, particularly for studies that meet the acceptable criteria for telehealth use.

We emphasize that with the mainstream use of telehealth becoming the new norm for conducting clinical visits; it has, in many ways, become interchangeable with in-person clinical visits. Even neuropsychological testing, a clinical practice anchored in standardized administration procedures has demonstrated patient acceptability and similar psychometric properties when performed via telehealth^12^. In a recent international survey deployed among the Movement Disorders Society that encompassed respondents across 40 countries, Hassan et al. reported a global increase in the use of telehealth in response to the pandemic^4^. These findings were consistent regardless of the country’s income categorization and prior use of telehealth. As stated by Dorsey et al., telehealth represents the “modern house-call” and should thus be considered synonymous to an in-person visit^6^. For example, in Canada, most provinces provide dedicated telehealth centers and personnel with access to teleconference softwares (e.g., Zoom) to deliver clinical care^4^. Thus, regulatory agencies should collectively standardize guidelines for the acceptability of telehealth visits for clinical research purposes to reduce site and sponsor burden of generating bespoke, site-specific solutions.

## Recommendations

To propose immediate action, we collectively urge IRBs to permit hybrid study visits that allow flexibility to conduct clinical assessments interchangeably in-person and remotely without the necessity for protocol amendments. The practicality, cost, and safety benefits of hybrid study designs far outweigh the risks of requiring participants to attend research visits in-person. Telehealth lowers barriers to reach those in rural and diverse communities, beyond large metropolitan centers where the vast majority of clinical trials are conducted. The motivation to offer telehealth services is further underscored by geographic areas where there is a shortage of highly trained Movement Disorder specialists, whereby assessments via new technologies may not only improve access to care, but offers clinicians with less familiarity with PD to make informed decisions on care. Thus, it is important to survey other countries to understand the needs, barriers, and learnings to maximize the benefits that telehealth may offer.

More importantly, while telehealth is currently used as a temporary solution to deliver healthcare during the pandemic, there should be considerations to continue the long-term use of telehealth as a tool to conduct clinical research. Incorporating telehealth options into future protocol designs would be a boon for rural and remote communities who currently miss out on novel treatments and are often excluded from clinical trials due to distance and requirements for in-person assessments. However, a major shortcoming for research studies is that not all assessments can be conducted via telehealth; in fact, remote or in-home services and the additional training required for technological platforms may increase operational costs for clinical trials. It is therefore important to acknowledge that sponsors must be willing to invest in the added infrastructure required to enable telehealth use for research studies. Sustainability of telehealth use in the US will also heavily depend upon compensation parity with in-person clinical visits by payers. We urge both regulators and institutions to widen the acceptability, use, and interchangeability of the use of telehealth for clinical research visits beyond the current pandemic climate.

Finally, as organizations leading the field of patient engagement in research and care, we urge IRBs, regulators, and institutions to overcome these hurdles to utilize telehealth in response to expressed community needs and priorities. As cited earlier in this Comment, our patient experience data indicate that the majority of PwP would prefer to continue telemedicine visits beyond the COVID-19 pandemic. Furthermore, integrating the patient community as equal partners in research and care model design and implementation is crucial for successful patient engagement and participation in clinical research. Therefore, if a field-wide guidance document for the use of telehealth to adapt in-person study visits and assessments is to be created, PwP and their care partners should be involved in the process.
